# First complex, then simple

**DOI:** 10.1186/1756-0381-7-13

**Published:** 2014-07-18

**Authors:** James D Malley, Jason H Moore

**Affiliations:** 1 Center for Information Technology, The National Institutes of Health, Bethesda, MD, USA; 2 Department of Genetics and Institute for Quantitative Biomedical Sciences, The Geisel School of Medicine, Dartmouth College, One Medical Center Dr, Lebanon, NH 03756, USA

## 

At the start of a data analysis project it is often suggested that the researcher look first at multiple simple models. That is, always begin with simple, one variable at a time analyses, such as multiple single-variable tests for association or significance. Then, later, somehow (how?) pull all the separate pieces together into a single comprehensive framework, an inclusive data narrative. For detecting true compound effects with more than just marginal associations, this is easily defeated with simple examples as has been recently highlighted in *BioData Mining*[1]. But more critically, it is looking through the data telescope from the wrong end.

It is our experience that first sifting, rigorously, carefully through complex data, as truly complex, is more efficient as a first step. After which the researcher can work to locate the simple models, the layers of the story. There are several interconnected issues to this problem and this approach.

First, as researchers ourselves we find it more efficient to use a fast filter to detect the presence of any signal, and then use these indications to fit smaller models. Many methods are available for doing this sensibly and quickly. Some of these are fully nonparametric statistical learning machines [2] and others are more parametric based schemes [3]. In any of these approaches, discovery is the initial goal.

Second, this approach is sometimes faulted on the grounds that results from a fast signal detector are impossible to interpret and hence are of little help. It is certainly true that learning machine output can be hard to understand if we stop with just the declaration of signal present, or signal not present. Yet many learning machine methods are available for sorting the results and then getting down to simple models.

Third, it is also true that multiple small or large models can be equally compelling and all more or less correct: the models of Nature are strongly underdetermined. Confirmation after discovery, after multiple disclosures should be reinforcing across domains and models. A single tiny best model is a rare event, not impossible, but also not efficiently considered as a likely and reachable endpoint.

Fourth, is this, that as terms, simple and complex are not simple and well-formed. Galileo believed that the arc of a water fountain was a catenary, but this is not correct: it is a parabola. On the other hand, the best supporting building arch is a catenary. And functionally a parabola is simpler than a catenary. Further, it was once thought that if planets did orbit around the Sun then they must do so in circles, since anything else, such as ellipses, would be too complex. This is not correct either. Note in these examples the detectors and data at the time were not sufficient to separate these models, confirming one, discounting another, and locating the best context for each model. But keeping all models available as working hypotheses might have promoted critical distinctions: consider using a catenary for building arches, but don’t patiently wait for a water fountain to assume the shape of a catenary. Galileo’s genius can be validated in other ways.

Summarizing, having a tool is often not the problem. It is knowing when to use it, and then which end to use.
